# Building Chemical Interface Layers in Functionalized Graphene Oxide/Rubber Composites to Achieve Enhanced Mechanical Properties and Thermal Control Capability of Tires

**DOI:** 10.3390/polym16162234

**Published:** 2024-08-06

**Authors:** Haixiang Jia, Xiaohe Miao, Zhiyi Zhang

**Affiliations:** 1 Department of Materials Science and Engineering, Shanxi College of Technology, Shuozhou 036000, China; jhx526955@163.com; 2 School of Materials Science and Engineering, North University of China, Taiyuan 030051, China; 15526691227@163.com

**Keywords:** graphene oxide, rubber–filler interactions, mechanical properties, heat build-up, green tires

## Abstract

With the rapid development of the transport industry, there is a higher demand for environmental friendliness, durability, and stability of tires. Rubber composites with excellent mechanical properties, abrasion resistance, and low heat generation are very important for the preparation of green tires. In this study, the all-aqueous phase process was initially employed to prepare 2-Amino-5-mercapto-1,3,4-thiadiazole (AZT) functionalized graphene oxide (AGO). Subsequently, modified graphene oxide/silica/natural rubber (AGO/SiO_2_/NR) composites were obtained through latex blending and hot press vulcanization processes. This method was environmentally friendly and exhibited high modification efficiency. Benefiting from the good dispersion of AGO in the latex and the cross-linking reaction between AGO and NR, AGO/SiO_2_/NR composites with good dispersion and enhanced interfacial interaction were finally obtained. AGO/SiO_2_/NR composites showed significantly improved overall performance. Compared to GO/SiO_2_/NR composites, the tensile strength (28.1 MPa) and tear strength (75.3 N/mm) of the AGO/SiO_2_/NR composites were significantly increased, while the heat build-up value (10.4 °C) and DIN abrasion volume (74.9 mm^3^) were significantly reduced. In addition, the steady-state temperature field distribution inside the tire was visualized by ANSYS finite element simulation. The maximum temperature of the prepared AGO/SiO_2_/NR was reduced by 18.2% compared to that of the GO/SiO_2_/NR tires. This strategy is expected to provide a new approach for the development of low energy consumption, environmentally friendly, and long-life rubber for tires.

## 1. Introduction

Rubber is used in the manufacturing of tires primarily because of its high elasticity, excellent wear resistance, good flex resistance, and low heat generation, which enable it to better fulfill the functions of tires. The main functions of tires are to transmit the driving force and traction during vehicle operation [1], absorb road shocks and vibrations, ensure vehicle stability and passenger comfort, and prevent vehicle damage. Natural rubber (NR), as a traditional polymer material, exhibits excellent elasticity, sealing properties, and wear resistance [2] and is widely used in tires, sealing components, rubber tubes, and other fields [3,4,5]. However, with the continuous advancement of industrial technology and increasing demands for product performance, traditional NR has struggled to meet the multifaceted requirements of certain applications. To endow rubber with the properties needed for tire manufacturing, large amounts of reinforcing fillers such as carbon black, silica (SiO_2_), and montmorillonite [6,7,8] are added to enhance its hardness, strength, wear resistance, and other properties. Unfortunately, traditional reinforcing fillers often require high dosages to meet usage requirements, significantly increasing production costs and processing difficulties. In recent years, nanofillers have received widespread attention in the preparation of rubber nanocomposites due to their exceptional properties. The low dosage of nanofillers can partially or fully replace high-content traditional reinforcing fillers, thereby reducing production costs and improving the processing performance of rubber [9].

Among different nanofillers, graphene has emerged as a promising reinforcing filler for polymer composites due to its exceptional thermal and electrical properties, as well as its large specific surface area [10,11,12]. However, the high cost of graphene increases the production cost of polymer composites [13]. Furthermore, the weak interfacial interaction between polar graphene and non-polar NR results in poor dispersion of the filler within the rubber matrix, leading to unsatisfactory reinforcement effects on the rubber. Graphene oxide (GO), as an important derivative of graphene, is cost-effective and can be reduced to graphene through reduction methods. More importantly, it possesses abundant oxygen-containing functional groups such as hydroxyl, carboxyl, and epoxy groups [14], which not only endow GO with good hydrophilicity and chemical activity but also provide possibilities for its further functionalization to improve compatibility with rubber. Currently, the reduction of GO mainly includes high-temperature reduction, chemical reduction, and electrochemical reduction methods [15,16,17]. However, after reduction, GO often faces issues such as the stacking of reduced GO (rGO) sheets due to strong interlayer interactions and reduced dispersibility in the rubber matrix.

In addition to focusing on the dispersion of graphene in polymers, it is also necessary to consider its interfacial interaction with polymers. Inspired by many high-performance polymer/nanocomposite materials, surface modifiers could impart active groups to nanofillers, which is beneficial for forming strong interfacial interactions through interfacial reactions, thereby enhancing the properties of polymers [18]. The methods for modifying GO mainly include non-covalent bond modification and covalent bond modification [19,20]. Fan et al. [21] used ionic liquids (IL, 1-allyl-3-methylimidazolium chloride) for non-covalent bond modification of GO. The double bonds in the IL enhanced the cross-linking reaction during the vulcanization process of the rubber composite at high temperatures, leading to enhanced interfacial interaction between the IL-modified GO and rubber macromolecules. Compared with covalent bonds, the interfacial interactions formed by ionic bonds and hydrogen bonds were weaker, which limited the reinforcing efficiency of GO and hindered the formation of organized graphene nanostructures in the rubber matrix. Therefore, many researchers have adopted covalent bond modification methods to enhance the interfacial interaction between graphene and rubber composites. Liu et al. and Zhao et al. [22,23] covalently modified GO fillers using silane coupling agents Si69 and KH590, respectively, to enhance the interfacial interaction between GO sheets and NR particles, which ultimately led to the enhancement of the mechanical properties of the composites. Recently, Zhong et al. and Cheng et al. [24,25] used vulcanization additives to load GO to obtain rubber composites with enhanced interfacial interactions. The interfacial enhancement was due to the fact that the thiol- or sulfur-containing functional groups in the modifier could form chemical bonds with the rubber molecular chains during the vulcanization process. Therefore, functionalizing GO through chemical or physical methods to improve its dispersion and interfacial interaction with the rubber matrix while reducing it has become key to preparing high-performance rubber composites. Unfortunately, most GO modification studies still face the issue of additional introduction of organic modifiers and organic pollutants, which can cause environmental pollution. The use of the all-water phase latex blending process minimized the environmental pollution caused by modifiers and organic solvents. The 2-Amino-5-mercapto-1,3,4-thiadiazole (AZT) modifier had both amino and thiol groups, so it could be easily soluble in water, which facilitated further modification of GO [26]. Compared with the mechanical blending method and melt blending method, the latex blending method offered advantages such as uniform blending of the filler with the latex liquid phase, better filler dispersion, reduced dust pollution, and environmental friendliness [27,28,29,30]. Furthermore, most research focuses more on enhancing mechanical properties while neglecting the damage caused by waste heat generated by viscoelasticity during actual tire use. This is detrimental to the development of green tires [31,32]. Developing green and environmentally friendly high-performance interfacial modifiers that do not require the introduction of excess organic matter is crucial for GO-reinforced green rubber tires. In recent years, SiO_2_ has been used to partially replace carbon black (CB) to simultaneously strengthen rubber and reduce rolling resistance [33].

In this study, functionalized GO (AGO) fillers were produced by covalently modifying GO with a vulcanization promoter (AMT). AGO fillers have both reinforcing and vulcanization-promoting functions without the need to introduce other redundant modifiers. The AGO/SiO_2_/NR composites were further obtained by latex blending and hot press vulcanization processes. AMT acted as a covalent bonding bridge between GO and NR to form strong chemical interfacial interactions at the interface of GO and NR molecular chains. As expected, the fabricated AGO/SiO_2_/NR composites exhibited high cross-linking density and excellent mechanical properties and heat build-up values. More importantly, the differences in the internal temperature of tread rubbers with different thermal control abilities were visualized by finite element simulation to predict the actual rolling effect of the tire when applied to tread rubber. AGO/SiO_2_/NR composites demonstrated a low steady-state temperature rise. This is expected to provide a new vision for the preparation of low-energy, high-performance graphene green tires.

## 2. Experimental Methods

### 2.1. Materials

Natural rubber latex (NRL, 60 wt.%) was purchased from Thai Rubber Economic and Trade Development Co, Wenzhou, China. Graphene oxide (GO, 1 wt.%) was provided by Changzhou Sixth Element Co, Changzhou, China. 2-Amino-5-mercapto-1,3,4-thiadiazole (AZT, 99%), hydroxysuccinimide (NHS, 98%), and 1-ethyl-3-(3-dimethylaminopropyl)carbodiimide (EDC, 98.5%) were obtained from Shanghai McLean Reagent Co, Shanghai, China. Other reagents and rubber vulcanization aids, including anhydrous ethanol, calcium chloride (CaCl_2_), zinc oxide (ZnO), stearic acid (SA), 2-(4-morpholinosulfanyl)-benzothiazole (NOBS), sulfur, Poly(1,2-dihydro-2,2,4-trimethylquinoline) (RD), and antioxidant 4020 were obtained from Aladdin, Shanghai, China.

### 2.2. Preparation of AGO Fillers

**Figure 1:**
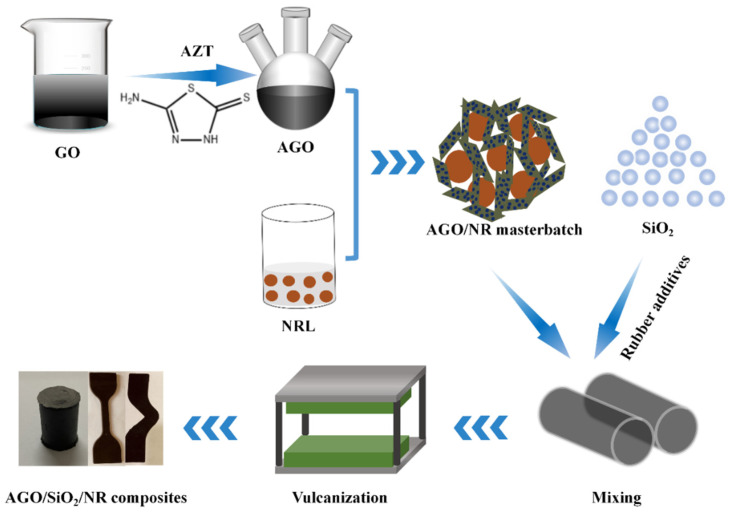
Schematic of the preparation of AGO/SiO_2_/NR composites.

AGO particles were prepared by a facile one-pot method as shown in Figure 1. Firstly, GO dispersion was diluted to 5 mg/mL by 20 min of ultrasonic cell crushing. Then, the EDC powder was added to the GO dispersion to activate the carboxyl groups. Subsequently, NHS and AZT powders were added to the GO dispersion during stirring. The mass ratio of GO/EDC/NHS/AZT was 1:0.02:0.02:1. After the above GO suspension was mechanically stirred in an ice-water bath for 12 h, it was washed by centrifugation with ethanol and water several times in sequence and then freeze-dried for 48 h to obtain AGO powder.

### 2.3. Preparation of AGO/SiO_2_/NR Composites

The AGO/NR masterbatch was first obtained using the latex co-precipitation method. Firstly, 5 mg/mL of AGO dispersion was added to the NRL. After being mechanically stirred for 15 min, 10 wt.% CaCl_2_ solution was used for complete flocculation. After removal of excess CaCl_2_ by extrusion and soaking, the AGO/NR masterbatch was dried in an oven at 60 °C until constant weight. The AGO/SiO_2_/NR compounds were obtained in two steps. In the first step, the AGO/SiO_2_/NR masterbatch and the rubber additives ZnO, SA, NOBS, RD, 4020, and SiO_2_ were mixed at 110 °C for 12 min in a compacting machine (Table 1, for details of the content of vulcanization additives). Then, the sulfur was mixed into the mix by shear force in a double-roll opener at 60 °C to obtain the final mix. Finally, the AGO/SiO_2_/NR vulcanized rubber was vulcanized in a hot press at 150 °C and 15 MPa for Tc_90_ (optimal vulcanization time).

Furthermore, in order to comparatively illustrate the significant contribution of the constructed chemical interfaces to the mechanical properties and thermal control ability of the NR composites, GO/SiO_2_/NR composites by addition of unmodified GO fillers and AZT/GO/SiO_2_/NR composites by addition of a simple blend of fillers with AZT and GO were obtained by employing the same process and were used as comparison samples. In order to make the name expression of the composites easier, GO/SiO_2_/NR, AZT/GO/SiO_2_/NR, and AGO/SiO_2_/NR composites have been denoted as S-1, S-2, and S-3, respectively, instead.

### 2.4. Characterizations

The chemical structures of GO, AZT, and AGO were analyzed by Fourier transform infrared (FTIR, IS50, Thermo Fisher, Waltham, MA, United States) with a scanning range of 4000–500 cm^−1^. The phase structures of the samples were detected by X-ray diffraction (XRD, 7200B, Haoyuan, Dandong, China) using Cu-Kα radiation at 40 kV and 40 mA. Raman spectroscopy (Raman, Invia, Renishaw, New Mills, UK) at a laser wavelength of 514.5 nm was used to characterize the changes in structural defects before and after GO modification. A field emission scanning electron microscope (FESEM, SU8010, Hitachi, Chiyoda, Japan) was used to characterize the micromorphology of the filler and NR composites. The filler powder needs to be pre-dispersed in ethanol and added dropwise to the wafer, while the NR composite samples need to be brittle at low temperatures in liquid nitrogen, and smooth planes were selected for patch observation. The atomic content and functional group distribution of GO were analyzed by X-ray photoelectron spectroscopy (XPS, NEXSA, Thermo Fisher). The amount of carbon residue and grafting rate of the samples were recorded using a thermogravimetric analyzer (TGA, Q50, TA New Castle, DE, USA), which was heated from 40 °C to 800 °C in nitrogen at 10 °C/min. Vulcanization characteristic parameters and curves of the NR composites were obtained using a Rubber Processing Analyzer (RPA, 8000-H, Gotech, Taichung City, Taiwan) at 150 °C test conditions. The dynamic temperature rise of the rubber was recorded using a dynamic compression heat generation tester (RH-3000N, Gotech) at a frequency of 30 Hz and a pressure of 1 MPa under the test conditions. The cylindrical rubber specimens of Φ17.8 mm × 25 mm used were vulcanized by specific molds. The thermal conductivity of the rubber was obtained using a steady-state thermal conductivity meter (DRL-III, Xiangtan, Xiangtan, China). Before the test, the thickness of Φ25 mm rubber sheet was measured by vernier calipers, and then thermal conductive silicone grease was uniformly applied on both sides. The cross-link density of the NR composites was calculated by the Flory–Reiner equation. The glass transition temperatures (Tg) of the NR composites were obtained by employing a differential scanning calorimeter (DSC, Mettler Toledo, Columbus, OH, USA) under an N_2_ atmosphere to cool down from room temperature to -80 °C at 5 ℃ /min and then to warm up to room temperature at 5 °C /min. The stress–strain curves were tested on a universal tensile testing machine (AI-7000, Gotech) with a tensile temperature of 25 °C and a tensile velocity of 500 mm/min. The hardness of the rubber composites was measured using a hardness tester (HS-A, Shanghai Precision Instrument and Equipment, Shanghai, China). The abrasion resistance of rubber materials was quantitatively evaluated using the DIN abrasion tester (GT-7012-D, Gotech).

## 3. Results and Discussion

### 3.1. Characterization of AGO

**Figure 2:**
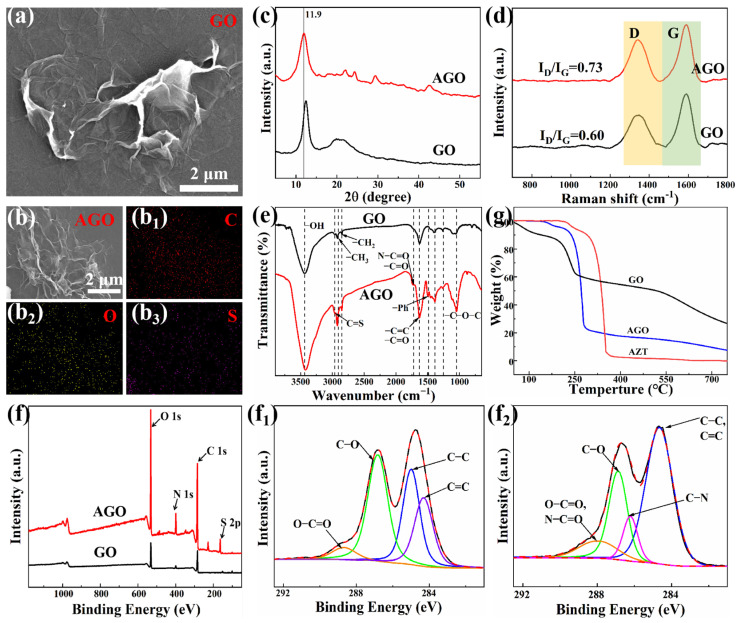
(**a**) SEM images of GO and (**b**) AGO; (**c**) XRD spectra, (**d**) Raman spectra, (**e**) FTIR spectraof GO and AGO fillers; (**f**) XPS full spectra of GO and AGO fillers and C1s of XPS spectra of GO (**f_1_**) and AGO (**f_2_**); (**g**) TG curves of GO and AGO fillers.

GO and AGO fillers were first observed by SEM. As shown in Figure 2a,b, GO presented a typical lamellar folded structure, and this structure provided sufficient surface area and reaction sites for the grafting of the AZT modifier. When GO was functionalized, the SEM morphology of AGO was similar to the typical folded structure of GO, indicating that the AZT-modified GO produced less effect on the structure of GO. From the EDS plots of the AGO filler, it was further found that a uniform distribution of S elements was detected on the surface of AGO, which indicated a uniform distribution of AZT molecules on AGO.

Figure 2c illustrates the XRD spectra of GO and AGO. It could be seen that the characteristic peak of the (001) crystal plane of GO was located at about 12.5°, and when GO was functionalized by AZT, the characteristic peak of its (001) crystal plane was shifted to about 11.9° in the direction of low angle [34]. According to Bragg’s equation, the lamellar spacing in GO increased from 0.356 nm to 0.373 nm. The increase in GO interlayer spacing was attributed to the spatial site resistance induced by AZT grafting at the GO edge. This also reflected the successful grafting of AZT onto GO. In addition, the characteristic peaks of AGO at 21.8°, 29.4°, and 42.7° might be related to AZT.

Raman spectroscopy is a powerful means to characterize the structural features of carbon materials and to obtain information about surface defects and functionalization of carbon materials. Usually, the value of I_D_/I_G_ could be used to respond to the degree of reduction of GO. From the Raman spectra of GO and AGO (Figure 2d), it could be seen that the I_D_/I_G_ values of GO and AGO fillers were 0.60 and 0.73, respectively. This was due to the fact that AZT was grafted on the edge of GO lamellae by chemical bonding, and the molecular structure of AZT determined a relative increase in sp^3^ hybridized molecular bonds in AGO, which indicated that AZT achieved an effective reduction of GO [35]. To further investigate the chemical structures of GO and AGO, FTIR spectra of GO and AGO were tested. The FTIR spectra are illustrated in Figure 2e. The epoxy group (C–O–C, 1053 cm^−1^), hydroxyl group (–OH, 3439 cm^−1^), carbonyl group (C=O, 1628 cm^−1^), and carboxyl group (–COOH, 1730 cm^−1^) could be observed from the spectra of GO [36,37]. The large number of oxygen-containing groups at the GO edges presented a negative charge balance in aqueous solution, which led to stable dispersion in water. Compared with the GO spectra, the AGO showed characteristic peaks at 1635 cm^−1^ (C=O stretching vibration), 2996 cm^−1^ (C=S sulfone group), 1496 cm−^1^ (-Ph), and 1703 cm^−1^ (N–C=O), which were associated with the AZT molecule. Since the C of the carbon ring on graphene had conjugated electrons, it could redshift the infrared absorption peak of the C–N bond. This indicated that AZT reacted chemically with GO. XPS was used to characterize the surface chemical composition of GO and AGO fillers. It can be noticed from Figure 2f that the peaks at 401 eV and 163 eV appeared for the first time in the spectrum of AGO, and they were N 1s and S 2p peaks. They originated from the covalently grafted AZT molecules on GO. Compared with the C1s spectrum of GO (Figure 2f_1_), it can be observed from Figure 2f_2_ that the peak intensities of the O–C=O and N–C=O bonds at 288.6 eV in the C1s spectrum of AGO increased, and a new C–N peak appeared at 286.2 eV [38]. This provides evidence for the effective reduction and covalent grafting of GO by AZT as a modifier. The thermal decomposition curves for GO, AGO, and AZT are shown in Figure 2g. For GO filler, the first weight loss was before 150 °C, which was due to the removal of adsorbed water. The second weight loss was from 150 °C to 500 °C, which was due to the decomposition of unstable oxygen functional groups on GO. The third stage occurred above 500 °C, which is mainly due to the decrease in thermal stability of the GO skeleton at the high-temperature stage. From the TG curve of AZT, it could be observed that a rapid decomposition occurred at 250 °C to 350 °C. Compared to the TG curves of GO, the weight loss of AGO filler functionalized with AZT in the second stage was higher than that of GO, which was caused by the thermal decomposition of the AZT modifier grafted on AGO. The mass fraction of AZT in AGO could be obtained from the difference in residual carbon weight to be about 59.3 wt.%.

### 3.2. Vulcanization and Cross-Linking Properties of AGO/SiO_2_/NR Composites

**Figure 3:**
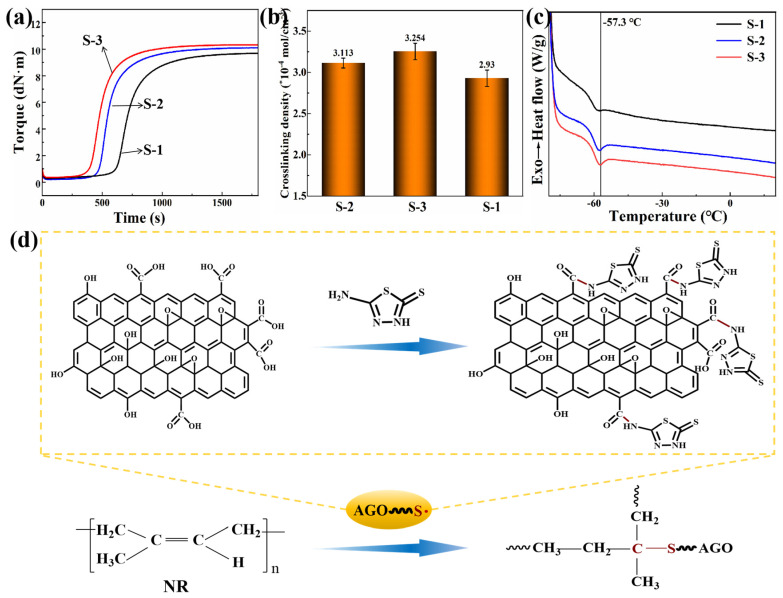
(**a**) Vulcanization characteristic curves, (**b**) cross-link density, and (**c**) DSC curves of NR composites with different interfacial structures. (**d**) Schematic diagram of the mechanism of cross-linking reaction between AGO and NR.

Due to the presence of sulfhydryl groups, the AZT molecule could form a chemical cross-linking bond with the NR molecular chain through a sulfhydryl–alkene click reaction. This reaction helped to build a stronger filler–matrix interface. This not only enhanced the interaction between the filler and the rubber matrix but might also have an effect on the vulcanization process of the rubber. The vulcanization characteristic curves of three different NR composites are presented in Figure 3a. Compared to the vulcanization time of S-1 composites, the vulcanization time of S-3 and S-2 composites was reduced. This was due to the adoption of sulfhydryl-containing organic molecules in the rubber vulcanization process, which could easily participate in the cross-linking reaction of the rubber molecular chain to form C–S bonds, thus increasing the overall vulcanization speed of the rubber. At the same time, it could be seen that the vulcanized rubber of S-3 composites had a greater torque, which was due to the formation of a chemical cross-linking interface between the molecular chains of AGO and NR through the sulfhydryl–alkene click reaction, which led to an increase in the resistance to deformation of the vulcanized rubber. Thanked to the chemical cross-linking interface that could be formed between AGO and NR, S-3 composites also exhibited a higher cross-linking density of 3.254 × 10^−4^ mol/cm^3^ (Figure 3b). The increased cross-linking density not only enhanced the mechanical properties of the rubber but might also have an effect on its thermal properties [39]. Furthermore, the glass transition temperature (T_g_) of the NR composites was obtained by DSC. It can be seen from Figure 3c that the glass transition temperature of S-3 composites was −57.3 °C, which was significantly higher than that of S-1 and S-2 composites. This was because the chemical cross-linking bond between the molecular chains of AGO and NR rubber increased the binding of the filler particles to the rubber molecular chains, which led to an increase in the T_g_ of the rubber. This indicated that the thermal stability of the rubber was improved, which was important in practical applications. A detailed schematic of the chemical reaction mechanism between AGO and NR is shown in Figure 3d [40,41,42].

### 3.3. Morphologies of NR/MGO Composites

During the rubber compounding process, the interaction force between the filler and the rubber matrix was affected by the processing conditions. When the filler was uniformly dispersed in the NR matrix and formed a strong interfacial bond with the NR, it enabled the NR to effectively resist the effects of external stresses and maintain the bond between the filler and the rubber matrix during processing. This interfacial layer was advantageous for rubber reinforcement because it could transfer the stress and prevent the slip between the filler and the rubber matrix, thus improving the mechanical properties of rubber.

**Figure 4:**
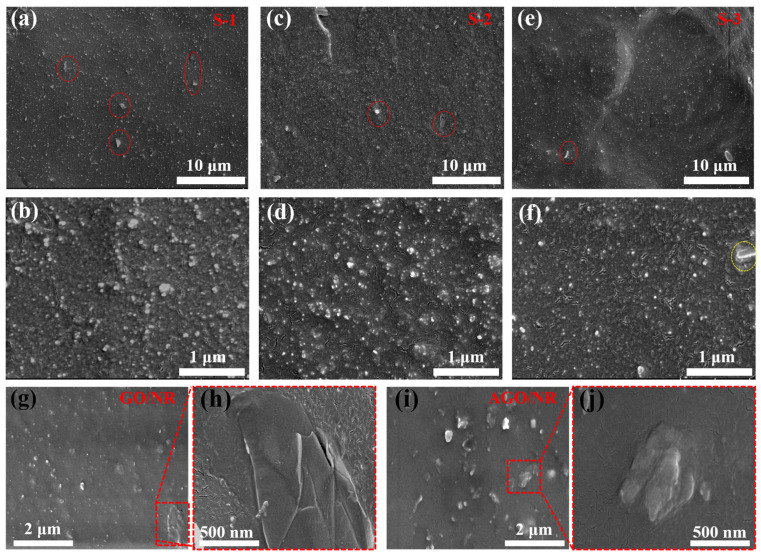
SEM images of (**a**,**b**) S-1, (**c**,**d**) S-2, (**e**,**f**) S-3, (**g**,**h**) GO/NR, and (**i**,**j**) AGO/NR composites.

From the SEM images of the brittle section of the S-1 composites (Figure 4a,b), it was observed that more agglomerates appeared, and it was more difficult to find the formation of an interfacial layer between the filler and NR. This was due to the fact that when the rubber was fractured, the lack of effective interfacial interaction between the filler and NR led to the pulling out of the filler from the rubber matrix. At the same time, the weak interaction between GO and NR led to a stronger interaction between GO and SiO_2_ particles, resulting in filler agglomeration. It can be found from the SEM images (Figure 4c,d) of the S-2 composites that compared with the GO/SiO_2_ composites, the addition of AZT did not effectively improve the agglomeration of the SiO_2_ particles due to the fact that the AZT modifier could not form a covalent linkage with GO and was only added by the time of blending. On the contrary, this situation was improved in S-3 composites. From the SEM of the AGO/SiO_2_/NR composites (Figure 4e,f), it can be observed that most of the AGO fillers were tightly connected with the NR matrix, and the SiO_2_ particles were uniformly dispersed in the rubber matrix. Due to the presence of AGO, it not only formed a good interfacial connection with the NR molecular chain but also prevented the agglomeration of SiO_2_ particles through the uniform dispersion of GO in NR due to its surface structural properties. In addition, in order to observe the interfacial difference between GO and AGO and NR more clearly, GO/NR and AGO/NR composites without SiO_2_ filler were prepared using the same process. As can be seen in the figure (Figure 4g,h), GO was more uniformly distributed in NR. However, due to the weak interaction between the oxygen-containing functional groups in the edge part of GO and NR, there was a large interface between some GO and NR. On the contrary, in the SEM images (Figure 4i,j) of AGO/NR composites, it can be observed that some of the AGO edges were more tightly connected with NR, which was due to the AZT on AGO acting as a bridge between the molecular chains of GO and NR and forming a strong covalent linkage cross-linking interface.

### 3.4. Heat Build-Up and Thermal Conductivity Properties of NR Composites

**Figure 5:**
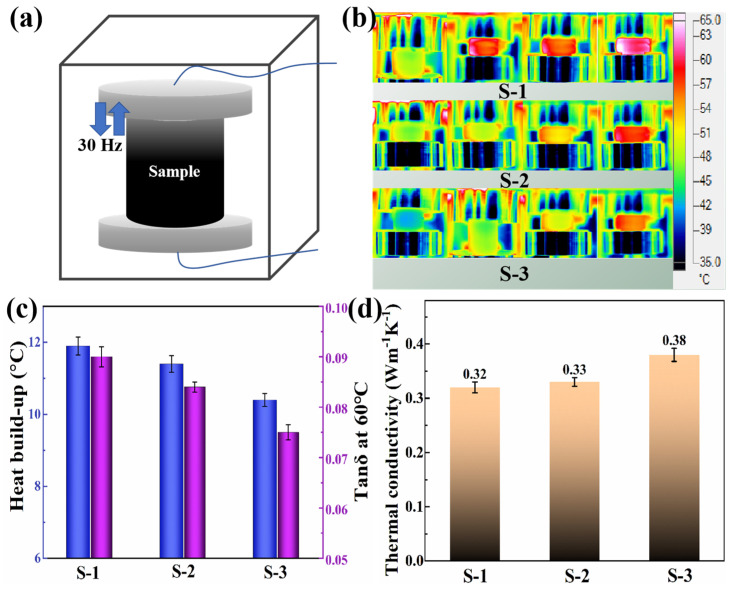
(**a**) Schematic diagram of compression fatigue test. (**b**) Infrared thermogram of the rubber cylindrical specimen during dynamic compression testing. (**c**) Compression fatigue heat build-up values and tanδ at 60℃ and (**d**) thermal conductivity properties of different NR composites.

During the dynamic deformation of the rubber, the interface between the filler and the rubber matrix undergoes relative motion, generating frictional heat. If the interfacial connection between the filler and the rubber matrix is weak, then this relative motion is more intense, and more frictional heat is generated. Conversely, when the interfacial connection between the filler and the rubber matrix is stronger, the filler is more stably anchored in the rubber’s cross-linked network, which reduces the relative motion and, therefore, the heat generated [43,44,45]. The rubber dynamic compression fatigue heat generation tester was used to track the temperature change in a rubber specimen as it underwent a dynamic compression deformation process. Its schematic is shown in Figure 5a. Combining the infrared thermograms (Figure 5b) and the final heat build-up values (Figure 5c) during the testing process, it was intuitively found that the AGO/SiO_2_/NR composites exhibited a low temperature variation (10.9 °C) during the testing process. In particular, the color of the IR thermograms of the S-3 composites was lighter compared to that of S-1 and S-2 composites, which indicated less temperature variation and lower heat generation value. This was mainly due to the fact that the grafted AZT molecules on AGO could react chemically with the NR molecular chains to form a stronger chemical interfacial interaction between GO and NR; this result was also confirmed by the tanδ result at 60 °C (Figure 5c). It not only fixes the AGO more stably in the cross-linked network of rubber to reduce the heat of slip friction between filler and rubber but also effectively reduces the heat of friction between fillers during dynamic stress. As a result, the S-3 composites exhibited less frictional heat and lower temperature rise values. The S-3 composites showed a 12.6% reduction in heat build-up value compared to the S-1 composites.

In addition, the thermal conductivity of NR composites with different interfacial structures was measured, and they are shown in Figure 5d. At the same filler content, the differences in thermal conductivity of the three NR composites were small. Among them, the thermal conductivity of S-3 composites was higher. This was due to the improved filler dispersion and interfacial interactions of the S-3 composites, which resulted in a reduction in the mean free range of phonons as heat transfer carriers and a reduction in scattering at the interfaces. As a result, the S-3 composites exhibited relatively good thermal conductivity, which would help to transfer the heat accumulated inside the rubber express to the outside environment.

### 3.5. Finite Element Simulation of Tire Temperature Field

In addition to enhancing the mechanical properties of the tread rubber to prevent mechanical damage to the tire, it is also necessary to reduce its thermal aggregation to prevent thermal damage to the tire. This is because when the tire is subjected to constant dynamic stress during driving, the tread rubber experiences severe heat generation and heat accumulation, resulting in high internal tire temperatures. The high temperature aggravates the aging of the tread rubber and the degradation of its mechanical properties, thus affecting its service life and safety. Therefore, the ability to control heat is particularly important for rubber composites used in tire tread.

**Figure 6:**
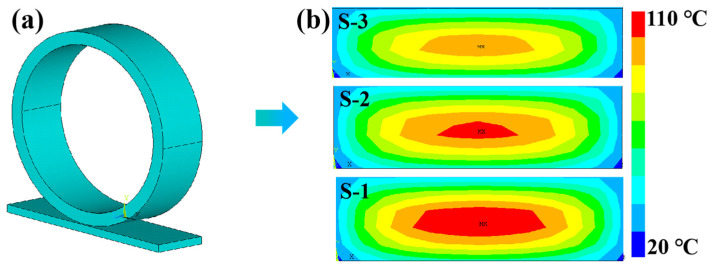
(**a**) Schematic of the finite element model of a solid tire. (**b**) Cross-sectional temperature field cloud images of tread rubber with different interfacial structures.

ANSYS finite element simulation was used to further evaluate the practical effectiveness of NR composites in tire applications [46,47,48]. Firstly, a finite element model of a solid tire and road surface was established, as shown in Figure 6a. Then, working conditions of a load of 2.5 t and speed of 60 km/h were applied to the tires to simulate the temperature field as the tires rolled to a steady state. The cloud images of steady-state temperature field distributions for NR tread rubber with three different interfacial structures are shown in Figure 6b, respectively. It can be visually observed that the internal temperature cloud image of S-3 tread rubber was darker in color when rolling to a steady state, which indicated that the temperature of its tire core was lower. The temperature cloud images of S-1 and S-2 tread rubbers were similar in color with a red cloud image, which reflected the higher internal temperature of their tread rubbers compared to that of S-3 tread rubbers. Compared to the maximum temperature of the S-1 tread rubber, the maximum temperature of the S-3 tread rubber was about 18.2% lower. This was mainly due to the stronger chemical interface of the S-3 tread rubber, which resulted in a lower heat build-up value. Moreover, the stronger chemical interfaces also resulted in lower interfacial phonon scattering and higher thermal conductivity of the S-3 composites, which allowed the heat generated inside the tire to be rapidly transferred to the air, hub, and road surface. Therefore, this confirmed that the thermal control ability of rubber composites could be effectively improved by constructing enhanced interfacial interactions between the filler and the matrix.

### 3.6. Mechanical Properties of NR Composites

**Figure 7:**
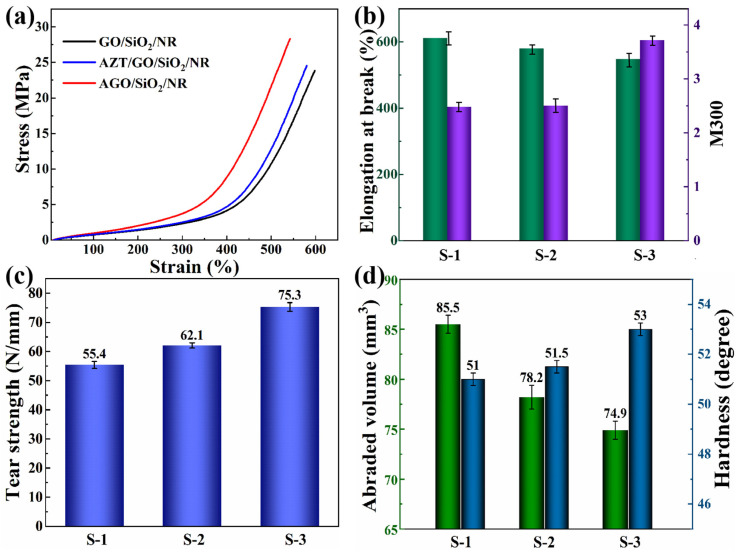
(**a**) Stress–strain curve, (**b**) Elongation at break and M300, (**c**) Tear strength, and (**d**) Abrasion properties of different NR composites.

In rubber composites, the state of dispersion of the filler directly affects its mechanical properties. Good dispersion ensures that the filler particles are uniformly distributed in the matrix, thus effectively transferring stress and preventing crack extension. In addition, the interaction between the filler and the matrix is a key factor in determining the mechanical properties of rubber composites. Strong interactions can ensure the stable presence of fillers in the matrix and effective stress transfer. The mechanical properties of NR rubber composites with different interfacial structures are shown in Figure 7.

Compared to the S-1 and S-2 composites, the S-3 composites had higher tensile and tear strengths and lower abrasion volume. This was mainly attributed to the fact that the incorporation of AGO effectively enhanced the interfacial interaction between the filler and the matrix, resulting in a more stable fixation of the AGO filler as a cross-linking point in the rubber matrix. As a result, when subjected to external forces, the S-3 composites were able to effectively transfer stresses and prevent crack extension, thus exhibiting higher tensile and tear strengths. It can be further observed from Figure 7b that the elongation at break of the S-3 composites was slightly reduced compared to other NR composites, which was attributed to the strong interactions between the AGO filler and the NR matrix limiting the ability of the rubber molecular chains to move during the tensile process. This led to the reduction of elongation at break. For tread rubber, the value of M300 was of more interest than the tensile strength as it is often used to represent the rubber modulus. The S-3 composites exhibited high modulus due to the excellent filler dispersion and interfacial interactions of the NR composites. DIN abrasion tests were based on the principle of rotational friction and simulated the abrasion of rubber materials in practical applications by allowing the rubber sample to rub against an abrasive wheel. In this process, the surface of the rubber sample was worn away by the friction. By measuring the mass change before and after wear and combining it with the density of the rubber sample, the abraded volume could be calculated. It was often used to indicate the abrasion resistance of rubber materials. Specifically, the smaller the abraded volume, the greater the abrasion resistance of the rubber. Thanks to the improved cross-link density and modulus, the S-3 composites showed lower abrasion volume and higher hardness, which suggested that the rubber composites had a good abrasion resistance. All these results suggested that the strategy of constructing a chemical interface between GO and rubber could simultaneously achieve excellent mechanical properties, low heat accumulation, and high abrasion resistance of tires. Therefore, this strategy is expected to provide a method for the preparation of graphene green tires.

## 4. Conclusions

In this study, AGO fillers with both reinforcing and vulcanization-promoting functions were prepared by covalent modification. Then, AGO/SiO_2_/NR composites were obtained by a latex blending and vulcanization process. The AGO was well dispersed in the NR matrix and formed strong interfacial interactions with the NR molecular chains through chemical cross-linking, which resulted in a significant improvement in the overall properties of the AGO/SiO_2_/NR composites. Compared with GO/SiO_2_/NR composites, the tensile strength of AGO/SiO_2_/NR composites was increased to 28.1 MPa, the tear strength was increased to 75.3 N/mm, the heat build-up value was reduced to 10.4 °C, and the DIN abrasion volume was reduced to 74.9 mm^3^. More importantly, finite element simulations of the solid tires further showed that the maximum temperature of the tires prepared with AGO/SiO_2_/NR tread rubber was reduced by 18.2% compared to that of GO/SiO_2_/NR tread rubber when reaching a steady state. Therefore, the comprehensive performance of rubber could be significantly improved by constructing a strong chemical cross-linking interface, and this strategy could provide a new idea for the development of high-performance green tires.

## Figures and Tables

**Table 1:** Formulae of the NR composites, phr.

Materials	Content (phr)
NR	100
GO, AGO	1.0
ZnO	5
S	2
SA	2
RD	2
4020 NA	2
NOBS	2
SiO_2_	30

## Data Availability

Data are contained within the article.
